# MIRIA: a webserver for statistical, visual and meta-analysis of RNA editing data in mammals

**DOI:** 10.1186/s12859-019-3242-2

**Published:** 2019-12-22

**Authors:** Xikang Feng, Zishuai Wang, Hechen Li, Shuai Cheng Li

**Affiliations:** 1 Department of Computer Science, City University of Hong Kong, Kowloon, Hong Kong; 2 Department of Biomedical Engineering, City University of Hong Kong, Kowloon, Hong Kong

**Keywords:** RNA editing, Visualization, Analysis, Webserver, Mammal

## Abstract

**Background:**

Adenosine-to-inosine RNA editing can markedly diversify the transcriptome, leading to a variety of critical molecular and biological processes in mammals. Over the past several years, researchers have developed several new pipelines and software packages to identify RNA editing sites with a focus on downstream statistical analysis and functional interpretation.

**Results:**

Here, we developed a user-friendly public webserver named MIRIA that integrates statistics and visualization techniques to facilitate the comprehensive analysis of RNA editing sites data identified by the pipelines and software packages. MIRIA is unique in that provides several analytical functions, including RNA editing type statistics, genomic feature annotations, editing level statistics, genome-wide distribution of RNA editing sites, tissue-specific analysis and conservation analysis. We collected high-throughput RNA sequencing (RNA-seq) data from eight tissues across seven species as the experimental data for MIRIA and constructed an example result page.

**Conclusion:**

MIRIA provides both visualization and analysis of mammal RNA editing data for experimental biologists who are interested in revealing the functions of RNA editing sites. MIRIA is freely available at https://mammal.deepomics.org.

## Background

RNA editing is defined as a critical post-transcriptional regulatory RNA-processing event (excluding RNA splicing) that generates an RNA transcript with a primary nucleotide sequence different from its gene. In mammals, the most common form of RNA editing, A-to-I RNA editing, is catalysed by the ADAR family of enzymes (Adenosine Deaminase that Acts on RNA) [1, 2], and this process leads to an A-to-G reading of the cDNA molecule [3, 4]. A-to-I RNA editing exists in the coding regions of many RNAs, including those encoding glutamate receptor subunits [5–7], the G protein-coupled serotonin 2C receptor [8] and the anti-genome of the hepatitis delta virus [9, 10]. The functional consequences of RNA editing in non-coding regions involve miRNA biogenesis [11], editing of miRNA seed regions [12] or target sequences within an mRNA [13] and nuclear retention [14]. Moreover, RNA editing has been shown to be associated with many diseases such as the autoimmune disorder Aicardi-Goutières syndrome [15], various viral infections [16] and different types of cancer [17].

Recently, increasing number of mammalian RNA editing databases have been published [18–22], there is a dire lack of online tools to perform mammalian RNA editing analysis. Therefore, we developed MIRIA (Mammalian RNA Editing Profiling and Interactive Analysis), a webserver which focuses on providing mammalian RNA editing statistics, genomic feature annotations, editing level calculations, genome-wide distributions of RNA editing sites, tissue-specific analyses and conservation analyses. Furthermore, we collected sequencing data of polyadenylated RNAs from eight organs (i.e., brain, heart, liver, spleen, lung, kidney, skeletal muscle, testis) across seven mammals (i.e., human, rhesus, rat, mouse, pig, cow, sheep) to test our webserver.

## Usage and implementation

### Data uploading and filtering

MIRIA is a new tool that was designed for RNA editing analysis in mammals. Users need to provide a compressed file (.zip) containing all RNA editing files from different mammals. The architecture of the zip file has two layers. The first layer contains all species folders (e.g., human, mouse, rat), and the second layer, which is inside each species folder, contains all RNA editing files of the species (e.g., tissue1.res, tissue2.res, tissue3.res). The format of the single RNA editing file (.res) is listed below (Table 1). By default, all RNA editing sites in the uploaded data are included to downstream analyses. Users have the option of filtering low-quality sites using the minimum supporting reads count cutoff and the minimum reads coverage cutoff on the uploading website interface. All sites that dissatisfy these criteria are excluded from downstream analyses.

**Table 1:** The format of a single RNA editing file

Chromosome	Location (0 base)	Location (1 base)	Type	Supporting information	Strand	AD:DP
chr1	1,266,631	1,266,632	TC	3	–	5:9
chr1	1,280,297	1,280,298	AG	2	+	2:2

Chromosome: Name of chromosome. Location (0/1 base): Position of the RNA editing site in the chromosome. Type: Type of the identified RNA editing site (e.g., AG means ‘A-to-G’, etc.). Supporting information: Number of supporting reads for this RNA editing site. Strand: Possible values are: ‘.’, ‘+’, and ‘-’, which corresponded to ‘uncertain’, ‘positive strand’, and ‘negative strand’, respectively. AD:DP: Number of reads that had this RNA editing site, and the number of reads that covered this location

### Percentage of all editing types

**Fig. 1:**
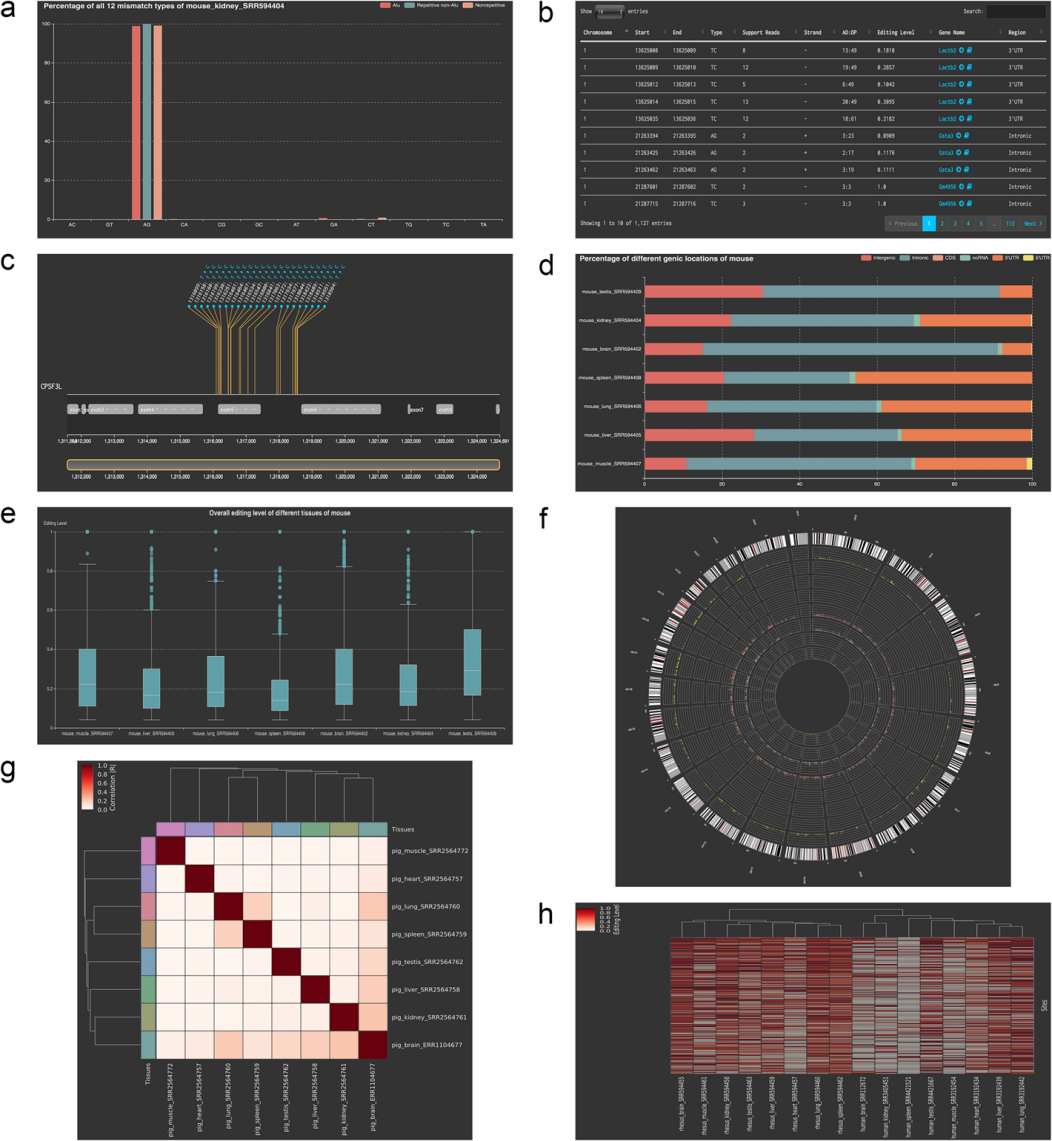
Example outputs of MIRIA. **a** Percentages of all 12 RNA editing types in Alu regions, repetitive non-Alu regions and non-repetitive regions. **b** Overview annotation table for a tissue. **c** Visualization interface showing all RNA editing sites within a gene. **d** Percentages of different genomic features across tissues in a species. **e** The overall RNA editing level across tissues in a species. **f** A Circos graph comparing the difference in the RNA editing numbers between different tissues at the genome-wide level. **g** Pearson correlations for the RNA editing levels of editing sites between various tissues in a species. **h** Heatmap showing the RNA editing levels of the top 200 conserved sites for human and other mammalian tissues. The hierarchical clustering dendrogram of tissues based on the correlations of the editing levels between tissues was appended at the top of the heatmap

Adenosine-to-inosine (A-to-I) editing is the most common form of editing in mammals. As such, the percentage of A-to-G editing, an indicator of A-to-I editing, is an important measurement to indirectly assess the detection accuracy of the RNA editing sites. Before calculating the percentage of A-to-G editing, we classified all editing sites into one of three categories, namely, those in Alu regions, those in repetitive non-Alu regions and those in non-repetitive regions. We performed the repeat region annotation using the RepeatMasker file downloaded from the UCSC Table Browser [23] . The percentages of all 12 editing types in each region were calculated separately. Specifically, the percentage of A-to-G editing was calculated based on the strand information of the uploaded data, which must be specified by users via the uploading website interface. For strand-specific data, only the A-to-G editing in the forward (+) strand and the T-to-C editing in the reverse (−) stand were regarded as A-to-G editing. For non-strand-specific data, all the A-to-G editing and the T-to-C editing were regarded as A-to-G editing. After the calculation, the results were represented as a bar chart (Fig. 1a).

### Genomic feature annotation

MIRIA can annotate RNA editing sites with a variety of useful genomic features. First, we used SnpEff [24], a genomic variant annotations tool, to annotate all RNA editing sites in the uploaded data. The annotation results were classified into six genomic clusters as follows: intergenic regions, intronic regions, CDS regions, ncRNAs (non-coding RNAs), 3′-UTRs and 5′-UTRs. Moreover, the corresponding gene name of each editing site was annotated. An overview annotation table could be accessed on the MIRIA web interface (Fig. 1b). Users could view all editing sites within one gene in an interactive visualization interface (Fig. 1c) by clicking on the gene name in the annotation table. Users could also explore the detailed information of the gene by clicking on the book icon adjacent to the gene name, which would directly link to the GeneCards page [25]. Besides the annotation table, MIRIA also generated an interactive bar chart to show the percentages of all the genomic features for each species (Fig. 1d).

### Overall editing level

To examine the editing level statistics of each tissue in the uploaded data, we determined the editing level of each RNA editing site as the ratio of the number of reads supporting this site to the number of reads covering this site. The overall editing level statistics of each tissue were displayed using a boxplot (Fig. 1e).

### Genome-wide distribution of RNA editing sites

For the statistical analysis of RNA editing sites on a genome-wide level, each chromosome was partitioned into contiguous 1-Mb windows, and the total number of RNA editing sites was calculated within each window. Thereafter, an interactive Circos graph was generated to compare the different RNA editing numbers between the different tissues on a genome-wide level (Fig. 1f).

### Tissue-specific RNA editing

To markedly improve the identification accuracy of the tissue-specific RNA editing sites, we removed the sites with a coverage less than 20. We then merged the RNA editing sites of all tissues in one species to one matrix and designated the RNA editing sites as rows and the tissues as columns. The ROKU R package [26] was applied to rank the RNA editing sites by their tissue specificity using the Shannon entropy. All sites satisfying two requirements, namely, that the editing level range (i.e., maximum editing level minus the minimum editing level) was larger than 0.1 and the Shannon entropy was less than 0.4 were reserved as tissue-specific RNA editing sites. The Shannon entropy cutoff could be adjusted by users on the data uploading interface. The absolute value of the Pearson correlation for the RNA editing levels of the tissue-specific sites between tissues was presented as a heatmap (Fig. 1g).

### Conserved RNA editing

To identify the conserved RNA editing sites in humans and other mammals, we adopted the UCSC LiftOver tool [23] to convert the genome position of each human reference to a mammalian reference. We also converted the genome position of other mammalian references to a human reference. The chain files of the human to the other mammalian references or the other mammals to the human reference were obtained from the UCSC download page. The RNA editing sites successfully converted on both turns (i.e., from the human to the other mammals and from the other mammals to the human) were reserved as conserved RNA editing sites. We used a heatmap to show the editing levels of the conserved RNA sites for tissues between the human and the other mammals. Moreover, the hierarchical clustering dendrogram of tissues based on the correlations of the RNA editing levels between tissues was appended at the top of the heatmap (Fig. 1h). By default, the heatmap only displayed the top 200 conserved RNA editing sites, which were sorted by the average editing level. Users had the option of adjusting the number of sites displayed in the heatmap on the data uploading interface.

### Results availability

After the submission of an analysis request, MIRIA returned a job ID to users, and users could check their job status with the ID. After the job completion, users could view the results by clicking on the “view result” link on the check job status page. All the results provided by MIRIA are publication ready. For the Circos graph, the PNG or SVG image file could be downloaded by clicking on the download button at the top of the results page. For the other graphs, the PDF or SVG image files are available. Moreover, the annotation table for each sample could also be downloaded as a tab delimited text file (.tsv) from the overview page of the job results interface.

### Webserver implementation

The MIRIA website was built using the Django Python Web framework [27] coupled with the MySQL database. The front-end interface was developed based on the Bootstrap open source toolkit [28]. The server-side data processing was supported by Docker [29]. The web interactive visualization graphs were developed using D3.js [30] and the ECharts [31] visualization library. The downloadable Circos graph was generated by the Circos software package, and the other graphs were produced using the ggplot2 R package and the Seaborn Python visualization library. MIRIA was published using the Apache Http server. The MIRIA website is freely available to all users, and there is no login requirement for accessing any of its features.

## Results

To evaluate the MIRIA webserver, we collected 55 RNA-seq datasets from seven mammals (i.e., human, mouse, rat, rhesus, pig, cow, sheep) as the test data. For each mammal, seven or eight tissue samples (i.e., brain, liver, lung, kidney, spleen, testis, heart, muscle) were used. The RNA-seq data were downloaded from the National Center for Biotechnology Information (NCBI) SRA database. The genomes and annotation files of the mammals were downloaded from the ENSEMBL database [32]. RepeatMasker files were downloaded from the UCSC table browser [23]. The reads were individually mapped to reference genomes using Hisat2 (v2.0.1) [33] with default parameters. The reference genomes used were as follows: human (hg38), mouse (mm10), rat (rn6), rhesus (rheMac8), pig (susScr3), cow (bosTau8) and sheep (oviAri3). The SAM files were sorted and converted to BAM files by Samtools (v1.2) [34] with default parameters. The RNA editing sites were identified using the “sprint_from_bam” program within SPRINT [35] with default parameters. We used the RNA editing sites data produced by SPRINT as the uploaded data for our webserver. The example results can be accessed by following the link https://mammal.deepomics.org/demo/.

## Conclusion

We developed the MIRIA to provide both visualization and analysis of mammal RNA editing data. MIRIA enables experimental biologists without any computational programming skills to perform a diverse range of analyses including RNA editing type statistics, genomic feature annotations, editing level statistics, genome-wide distribution of RNA editing sites, tissue-specific analysis and conservation analysis. For every analysis, the result is presented with a visualized graph and can be downloaded as a publication-ready format. In general, with the functions of the MIRIA designed for mammal RNA editing data, we believe that this webserver will be a valuable resource for experimental biologists who are interested in revealing the functions of RNA editing sites.

## Availability and requirements

**Project name:** MIRIA

**Project home page:** https://mammal.deepomics.org/

**Operating system(s):** Platform independent

**Programming language:** Python

**Other requirements:** Chrome, Safari, Firefox or IE

**License:** GNU GPL

**Any restrictions to use by non-academics:** None

## Supplementary information

**Additional file 1: Table S1.** The data source from NCBI.

## Data Availability

All the data used in our research were downloaded from the National Center for Biotechnology Information (https://www.ncbi.nlm.nih.gov/). And the Sequence Read Archive (SRA) id can be found at Additional file 1: Table S1.

## Abbreviations

ADAR: Adenosine Deaminase that Acts on RNA
CDS: Coding DNA Sequence
ncRNAs: non-coding RNAs
SRA: Sequence Read Archive
UTR: Untranslated Region
