# Supplementary material for: MIRIA: a webserver for statistical, visual and meta-analysis of RNA editing data in mammals
Source: BMC Bioinformatics. 2019 Dec 22;20(Suppl 24):596. doi: 10.1186/s12859-019-3242-2 (PMC6923819; doi:10.1186/s12859-019-3242-2)
Supplement: Supplementary file 1 — Additional file 1: Table S1. The data source from NCBI. [file 12859_2019_3242_MOESM1_ESM.docx]

**Supplementary Materials**

MIRIA: a webserver for statistical, visual and meta-analysis of RNA editing data in mammals

**Authors**

Xikang Feng^1†^, Zishuai Wang^1†^, Hechen Li^1^ and Shuaicheng Li^1,*^

**Affiliation**

^1^ Department of Computer Science, City University of Hong Kong, Kowloon, Hong Kong, ^2^ Department of Pig Genomic Design and Breeding, Agricultural Genome Institute at Shenzhen, Chinese Academy of Agricultural Sciences, Shenzhen 518124, China.

^†^ These authors contributed to this work as first authors.

**Table S1**. The data source from NCBI.

| **Species** | **Tissue** | **SRA ID** |
| --- | --- | --- |
| **Cow** | **brain** | **SRR594473** |
|  | **heart** | **SRR594475** |
|  | **kidney** | **SRR594476** |
|  | **liver** | **SRR594477** |
|  | **lung** | **SRR594478** |
|  | **muscle** | **SRR594479** |
|  | **spleen** | **SRR594480** |
|  | **testis** | **SRR594481** |
| **Human** | **brain** | **SRR112672** |
|  | **heart** | **SRR3192434** |
|  | **kidney** | **SRR3405451** |
|  | **liver** | **SRR3192439** |
|  | **lung** | **SRR3192442** |
|  | **muscle** | **SRR3192454** |
|  | **spleen** | **SRR4421521** |
|  | **testis** | **SRR4421667** |
| **Mouse** | **brain** | **SRR594402** |
|  | **kidney** | **SRR594404** |
|  | **liver** | **SRR594405** |
|  | **lung** | **SRR594406** |
|  | **muscle** | **SRR594407** |
|  | **spleen** | **SRR594408** |
|  | **testis** | **SRR594409** |
| **Pig** | **brain** | **ERR1104677** |
|  | **heart** | **SRR2564757** |
|  | **kidney** | **SRR2564761** |
|  | **liver** | **SRR2564758** |
|  | **lung** | **SRR2564760** |
|  | **muscle** | **SRR2564772** |
|  | **spleen** | **SRR2564759** |
|  | **testis** | **SRR2564762** |
| **Rat** | **brain** | **SRR594428** |
|  | **heart** | **SRR594430** |
|  | **kidney** | **SRR594431** |
|  | **liver** | **SRR594432** |
|  | **lung** | **SRR594433** |
|  | **muscle** | **SRR594434** |
|  | **spleen** | **SRR594435** |
|  | **testis** | **SRR594436** |
| **Rhesus** | **brain** | **SRR594455** |
|  | **heart** | **SRR594457** |
|  | **kidney** | **SRR594458** |
|  | **liver** | **SRR594459** |
|  | **lung** | **SRR594460** |
|  | **muscle** | **SRR594461** |
|  | **spleen** | **SRR594462** |
|  | **testis** | **SRR594463** |
| **Sheep** | **brain** | **ERR489218** |
|  | **heart** | **ERR489268** |
|  | **kidney** | **ERR489260** |
|  | **liver** | **ERR489264** |
|  | **lung** | **ERR489282** |
|  | **muscle** | **ERR489117** |
|  | **spleen** | **ERR489250** |
|  | **testis** | **ERR489224** |
